# *motifbreakR*: an R/Bioconductor package for predicting variant effects at transcription factor binding sites

**DOI:** 10.1093/bioinformatics/btv470

**Published:** 2015-08-12

**Authors:** Simon G. Coetzee, Gerhard A. Coetzee, Dennis J. Hazelett

**Affiliations:** 1 ^1^Bioinformatics and Computational Biology Research Center, Cedars-Sinai Medical Center, Los Angeles, CA, USA and; 2 ^2^Department of Urology and Preventive Medicine, USC Norris Comprehensive Cancer Center, Los Angeles, CA, USA

## Abstract

**Summary:** Functional annotation represents a key step toward the understanding and interpretation of germline and somatic variation as revealed by genome-wide association studies (GWAS) and The Cancer Genome Atlas (TCGA), respectively. GWAS have revealed numerous genetic risk variants residing in non-coding DNA associated with complex diseases. For sequences that lie within enhancers or promoters of transcription, it is not straightforward to assess the effects of variants on likely transcription factor binding sites. Consequently we introduce *motifbreakR*, which allows the biologist to judge whether the sequence surrounding a polymorphism or mutation is a good match, and how much information is gained or lost in one allele of the polymorphism or mutation relative to the other. *MotifbreakR* is flexible, giving a choice of algorithms for interrogation of genomes with motifs from many public sources that users can choose from. *MotifbreakR* can predict effects for novel or previously described variants in public databases, making it suitable for tasks beyond the scope of its original design. Lastly, it can be used to interrogate any genome curated within bioconductor.

**Availability and implementation:** https://github.com/Simon-Coetzee/MotifBreakR, www.bioconductor.org.

**Contact:** dennis.hazelett@cshs.org

## 1 Introduction

Transcription factor binding sites (TFBS) are typically short DNA sequence motifs that facilitate binding of a specific transcription factors via protein–DNA interactions (Stormo, 2000). There are some software tools that facilitate the scoring of non-coding variants with respect to either predefined or user-specified motifs. RegulomeDB, HaploReg and FunSeq each enable assessment of the effects of single nucleotide variants on predicted binding sites (Boyle *et* *al.*, 2012; Khurana *et al.*, 2013; Ward and Kellis, 2012). Each of these packages has strengths, but does not provide the analysis independent of its other functions. Many users generate their own motifs, but users are limited to built-in motif collections and functions. The functions are largely unavailable to non-human data sets. We hereby introduce an R/bioconductor software package called *motifbreakR* that addresses these major concerns. Implementation in R has the advantage of universality: R and bioconductor are widely used for bioinformatics and well supported across different platforms including Galaxy (Tenenbaum, 2015).

## 2 Features

### 2.1 Germline or somatic variants

**Fig. 1.**
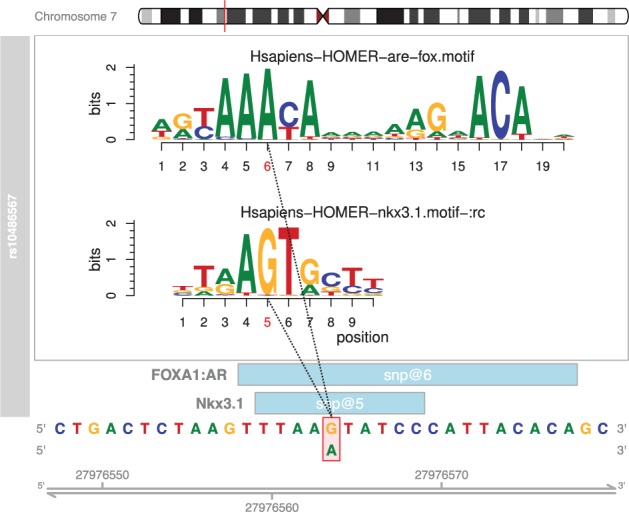
Example of *motifbreakR* output from *plotMB* function for a previously published SNP (Hazelett *et al*., 2014). Genomic sequence and coordinates are at the bottom of the display; the positions of the matches represented (light blue boxes). The position of the SNP within the motif is indicated with red bounding box and alternate allele below, and as red text on the motif logo position bar above. The motif logos generated from *motifstack* are shown above using the color conventions of the genomic sequence below

Single nucleotide polymorphisms (SNPs) can be generated from another R package such as FunciSNP (Coetzee *et* *al.*, 2012), or read in directly from .bed or .vcf files. The SNPs then need to be converted to GRanges objects. This is accomplished within *motifbreakR* in one of two ways, via the snps.from.rsid and snps.from.file functions. *MotifbreakR* will produce a GRanges table with match statistics describing the percent of maximum score for a matrix for both alleles of the SNP, the matrix values for each allele (useful for determining the severity of the disruption), the strand, whether the disruption is strong or weak. The package also includes a plotting function based on the *gviz* and *motifstack* packages as shown for SNP rs10486567 in Figure 1.

### 2.2 Comprehensive positional weight matrices

Once a SNP list is converted to GRanges, selection of PWMs proceeds by subsetting from the *MotifDb* package or from one of the included motifList-compatible libraries. Any motifList object can be passed to *motifbreakR.* *MotifDb* provides access to ScerTF (Spivak and Stormo, 2012), FlyFactorSurvey (Zhu *et* *al.*, 2011), hPDI (Xie *et* *al.*, 2010), all the selex-generated motifs from (Jolma *et* *al.*, 2013), UniPROBE (Newburger and Bulyk, 2009) and JASPAR (Stormo, 2000). In addition to these, we have included motifs from Encode (Kheradpour and Kellis, 2014), Homer (Heinz *et* *al.*, 2010), Factorbook (Wang *et* *al.*, 2012) and HOCOMOCO (Kulakovskiy *et* *al.*, 2013). Importantly, *MotifbreakR* generates pseudocounts based on the number of observations listed for each motif in these databases, but defaults to 20 observations when this information is lacking.

### 2.3 Choice of algorithms

There are three algorithms accessible via the ‘method’ argument. The first is the standard sum of log probabilities (method = ‘log’). The second and third are the weighted sum and information content methods (method = ‘default’ and method = ‘ic’). The default was previously described (Hazelett *et* *al.*, 2014); the ic method is based on relative entropy but renders very similar conclusions. The methods are extensively documented in the vignette. For all three methods, *motifbreakR* scores and reports the reference and alternate alleles of the sequence, and the effect (strong, weak or neutral). The match scores are scaled as a fraction of scoring range [0,1] of the motif matrix.

### 2.4 Extensible to model organisms

It is straightforward within the *motifbreakR* package to specify any of the research model organism genomes in *BSgenome* (currently 22 in number), including mouse, zebrafish, and fruit fly. This may be done both at the level of reading in SNP lists and during invocations of *motifbreakR.* To our knowledge, no other software currently offers this functionality.

## 3 Conclusion

In principle, a SNP label contains all the information necessary to characterize a variant or mutation, since it points to information in external databases somewhere. Our package makes it possible to rapidly explore TFBS disruptions for a large number of SNPs within the R framework, with no need to install third party software or massage arcane output files for downstream analysis. Although the intention is to study the relationship of human variation to disease, use of *motifbreakR* is not limited to this application. Indeed, one may access any genome in *BSgenome*, and query it with custom SNPs and PWMs, or specify organism-specific sets of PWMs from *MotifDb.* *motifbreakR* uses a highly efficient information content-based algorithm for discriminating between truly disruptive variants versus neutral. Because *motifbreakR* is designed to work with the existing bioconductor framework, we believe it to be the most flexible and extensible package available for this type of analysis.
